# Human Globozoospermia-Related Gene *Spata16* Is Required for Sperm Formation Revealed by CRISPR/Cas9-Mediated Mouse Models

**DOI:** 10.3390/ijms18102208

**Published:** 2017-10-21

**Authors:** Yoshitaka Fujihara, Asami Oji, Tamara Larasati, Kanako Kojima-Kita, Masahito Ikawa

**Affiliations:** 1 Research Institute for Microbial Diseases, Osaka University, Suita, Osaka 565-0871, Japan; fujihara@biken.osaka-u.ac.jp (Y.F.); oji-a@cdb.riken.jp (A.O.); tamara@biken.osaka-u.ac.jp (T.L.); kojima0208@patho.med.osaka-u.ac.jp (K.K.-K.); 2 Graduate School of Pharmaceutical Sciences, Osaka University, Suita, Osaka 565-0871, Japan; 3 RIKEN Center for Developmental Biology, Kobe, Hyogo 650-0047, Japan; 4 School of Pharmaceutical Sciences, Osaka University, Suita, Osaka 565-0871, Japan; 5 Graduate School of Medicine, Osaka University, Suita, Osaka 565-0871, Japan; 6 The Institute of Medical Science, The University of Tokyo, Minato-ku, Tokyo 108-8639, Japan

**Keywords:** genome editing, male infertility, mouse model, point mutation, spermatogenesis, testis

## Abstract

A recent genetic analysis of infertile globozoospermic patients identified causative mutations in three genes: a protein interacting with C kinase 1 (*PICK1*), dpy 19-like 2 (*DPY19L2*), and spermatogenesis associated 16 (*SPATA16*). Although mouse models have clarified the physiological functions of *Pick1* and *Dpy19l2* during spermatogenesis, *Spata16* remains to be determined. Globozoospermic patients carried a homozygous point mutation in *SPATA16* at 848G→A/R283Q. We generated CRISPR/Cas9-mediated mutant mice with the same amino acid substitution in the fourth exon of *Spata16* to analyze the mutation site at R284Q, which corresponded with R283Q of mutated human SPATA16. We found that the point mutation in *Spata16* was not essential for male fertility; however, deletion of the fourth exon of *Spata16* resulted in infertile male mice due to spermiogenic arrest but not globozoospermia. This study demonstrates that *Spata16* is indispensable for male fertility in mice, as well as in humans, as revealed by CRISPR/Cas9-mediated mouse models.

## 1. Introduction

Infertility is a major health concern around the world. One cause of male infertility is abnormal sperm formation due to genetic and/or epigenetic problems. Recent genetic studies of infertility patients identified causative mutations in three genes: a protein interacting with C kinase 1 (*PICK1*), dpy 19-like 2 (*DPY19L2*), and spermatogenesis associated 16 (*SPATA16*) [1,2,3]. These mutations were considered to be responsible for the infertility syndrome globozoospermia, caused by the malformation or loss of the sperm acrosome accompanied by an abnormal nuclear shape, as well as an abnormal arrangement of the sperm mitochondria. These abnormalities occur during spermatogenesis, especially during acrosome formation. The acrosome is a membrane-bound cap-like structure that covers the anterior portion of the sperm nucleus. In the testis, acrosome biogenesis has several steps: protein processing in the endoplasmic reticulum, vesicle trafficking from the Golgi apparatus, vesicle fusion, and the interaction of the acrosomal membrane with the nuclear membrane [4]. Globozoospermia is a rare infertility syndrome that shows severe abnormal sperm morphology. Although an intracytoplasmic sperm injection (ICSI) is a treatment option for infertile patients, globozoospermic spermatozoa show quite low rates of fertilization due to reduced oocyte activation ability [5]. The molecular mechanism of acrosome biogenesis and the mechanism that leads to globozoospermia remain to be determined.

PICK1 is a peripheral membrane protein that is ubiquitously expressed in mouse and human tissues [6,7]. PICK1 interacts with a number of membrane proteins and lipid molecules and regulates protein trafficking in the central nervous system [8]. Although *Pick1* gene knockout (KO) mice were viable and showed no gross developmental defects [9], KO males were infertile with abnormal sperm heads reminiscent of globozoospermia [10]. PICK1 localizes to Golgi-derived proacrosomal vesicles and is involved in vesicle trafficking from the Golgi apparatus to the sperm acrosome in a mouse testis [10]. A homozygous missense mutation (G198A) in the thirteenth exon of the *PICK1* gene was identified from globozoospermic patients in China [2]. This autosomal recessive genetic mutation in *PICK1* was responsible for globozoospermia in humans.

DPY19L2 is a testis-specific transmembrane protein [11] localized to the inner nuclear membrane of mouse spermatids [12]. Globozoospermic patients were found to carry a 200 kb homozygous deletion encompassing the entire *DPY19L2* locus [11,13]. A large cohort analysis of globozoospermic patients identified novel mutations and deletions of the *DPY19L2* locus [1,14,15,16,17]. Moreover, the *Dpy19l2* gene in KO mice also reproduced the infertile phenotype of globozoospermia with acrosomeless round-headed spermatozoa [12]. Therefore, *DPY19L2* was found to be a major causative gene of globozoospermia in humans.

SPATA16 (previously named NYD-SP12) is highly expressed in human testes and contains a conserved tetratricopeptide repeat (TPR) domain which is known to mediate protein-protein interactions [18]. SPATA16 is localized to the Golgi apparatus and to the proacrosomal vesicles, which fuse to form the acrosome during spermiogenesis [19]. A homozygous mutation (848G→A, R283Q) in the fourth exon of *SPATA16* was identified in three affected brothers from an Ashkenazi Jewish family. The fourth exon of *SPATA16* encodes the C-terminus of the TPR domain; the authors reported that the 848G→A mutation causes the disruption of the TPR domain due to fourth exon skipping [3]. However, this mutation was not found in 29 other patients with globozoospermia in Europe and North Africa [3]. These results may suggest that *SPATA16* is not the main cause of globozoospermia in humans [4].

In gene KO mouse studies, various genes (*Atg7* [20], *Csnk2a2* [21], *Dpy19l2* [12], *Gba2* [22], *Golga2* [23], *Gopc* [24], *Hrb* [25], *Hsp90b1* [26], *Mfsd14a* [27], *Pick1* [10], *Sirt1* [28], *Slc9a8* [29], *Smap2* [30], *Spaca1* [31], *Tmf1* [32], *Vps54* [33], *Zpbp1* [34]) were found to be associated with globozoospermia [4]. Although almost all genes (14 out of 17 genes) were ubiquitously expressed, these genes were unexpectedly responsible for male fertility. Only three genes, *Dpy19l2*, *Spaca1*, and *Zpbp1*, were testis-specific in mice and humans. However, there are no reports of causative mutations of *ZPBP1* and *SPACA1* in infertile patients [4,35]. Although genetic studies of human *SPATA16* have been reported, the physiological role of mouse *Spata16* has not been determined.

In this study, we generated *Spata16* mutant mice: a targeted point mutation (851G→A, R284Q) and a 781-bp deletion by CRISPR/Cas9. The targeted point mutation (851G→A, R284Q) was designed at the corresponding site (848G→A, R283Q) of infertile patients with globozoospermia as previously reported [3]. The 781-bp deletion of the *Spata16* locus caused the mis-translation of the fourth exon that encodes the C-terminus of the TPR domain.

## 2. Results

### 2.1. Testis-Specific Expression and Protein Sequence Alignment of Mouse SPATA16

**Figure 1:**
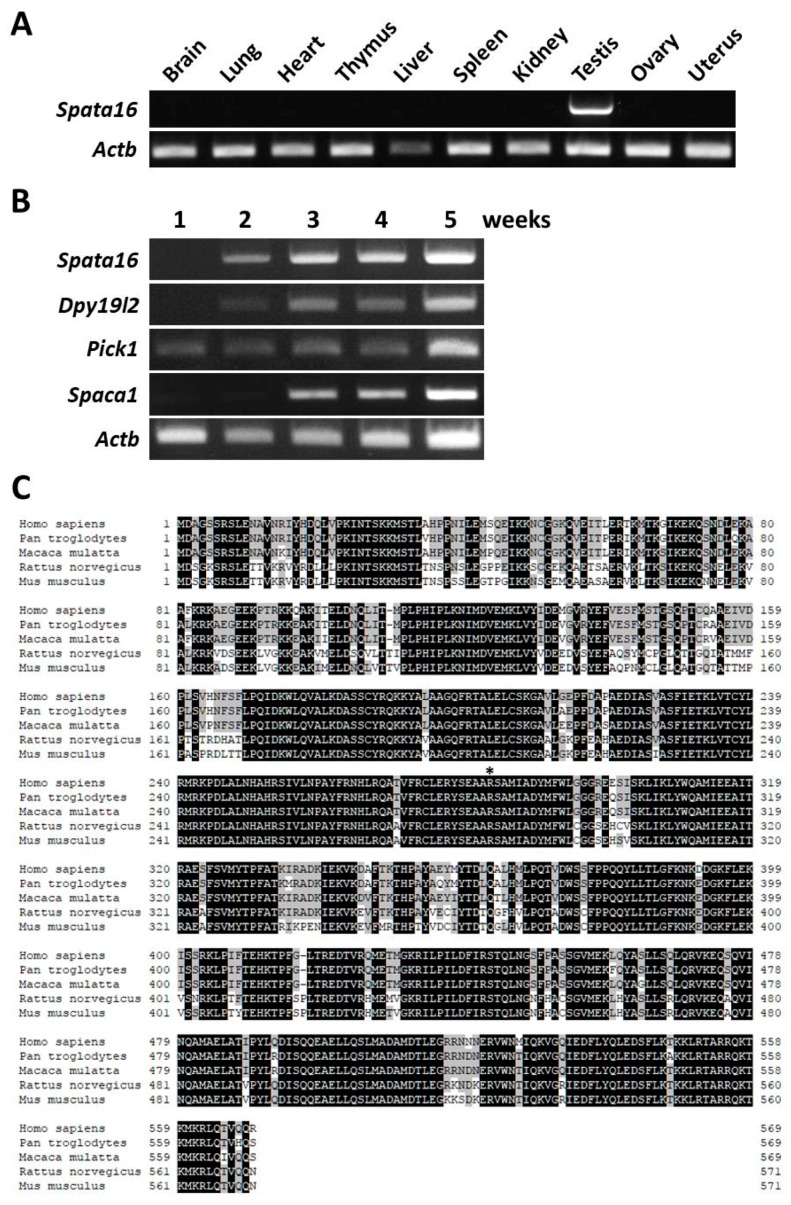
Characterization of mouse *Spata16*. (**A**) Testis-specific expression of *Spata16* by multi-tissue RT-PCR analysis. The expression of mouse *Spata16* was examined by RT-PCR using RNA isolated from various organs. *Spata16* was only detected in the testis. The *Actb* gene was used as an expression control; (**B**) RT-PCR analysis of *Spata16* and other globozoospermia-related genes in the mouse testis. *Spata16* and *Dpy19l2* were first expressed in a two-week-old testis. The onset of *Spata16* expression occurred prior to that of *Spaca1* encoding a sperm acrosomal protein; (**C**) Sequence similarity of SPATA16 protein in mammals. Black indicates a match in all species. Gray indicates a match among at least three species. The 283rd arginine residue of human SPATA16 as previously reported [3] is indicated by an asterisk. 79% of the SPATA16 amino acid sequence is identical between humans and mice.

The expression of mouse *Spata16* in various organs was examined by RT-PCR analysis. *Spata16* was exclusively expressed in mouse testes (Figure 1A), as previously reported in humans [18]. Next, the onset of *Spata16* expression was examined by RT-PCR and compared with that of globozoospermia-related genes: *Dpy19l2* [12], *Pick1* [10], and *Spaca1* [31]. Although *Pick1* was first detected in a one-week-old testis, *Spata16* and *Dpy19l2* were expressed in a two-week-old testis (Figure 1B). *Spaca1* was detected in a three-week-old testis during the acrosome formation stage [31]. These data showed that mouse *Spata16* is a testis-specific gene and begins expression prior to acrosomal biogenesis. Figure 1C shows the sequence similarity of the SPATA16 protein among species. SPATA16 is highly conserved in mammals, from mice to humans. More than 90% of the TPR domain is identical between mice and humans [3]. The point mutation (848G→A) causing a single amino acid substitution (R283Q) at the C-terminal end of the TPR domain was found in globozoospermic patients [3]. This amino acid, R284, is also conserved in mice (asterisk in Figure 1C).

### 2.2. Targeted Point Mutation and Deletion of Spata16 by CRISPR/Cas9

**Figure 2:**
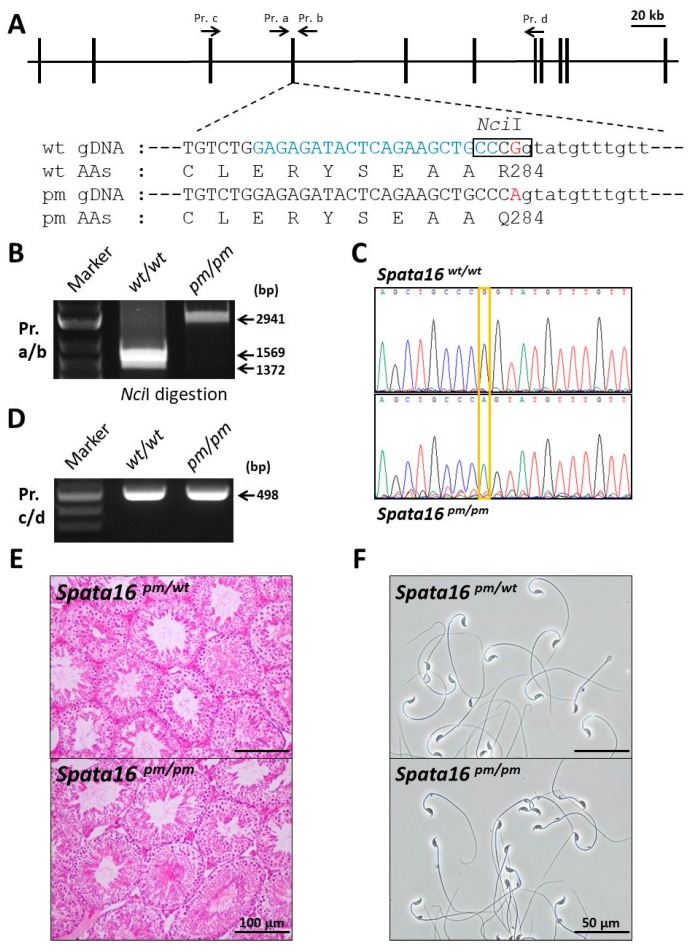
Targeted point mutation (851G→A, R284Q) of *Spata16* by CRISPR/Cas9. (**A**) Targeting scheme of the point mutation (851G→A, R284Q) in the fourth exon of mouse *Spata16*. *Spata16* consists of eleven exons. The point mutation was introduced into mouse ES cells using the HDR donor plasmid with 0.5 kb homology arms. Blue indicates the sgRNA sequence. Red indicates a G→A mutation at the last nucleotide (851st coding exon) of the fourth exon of *Spata16*. Capital and small letters indicate nucleotides of exons and introns, respectively; (**B**) The genotyping of point-mutant mice by *Nci*I digestion. The *Nci*I recognition site (5′-CCCGG-3′) was disrupted due to the G→A mutation of the fourth exon. In wild-type mice, two bands (1372 bp and 1569 bp) were detected after *Nci*I digestion; (**C**) Direct sequencing of PCR products in *Spata16*^pm/pm^ mice. The G→A mutation at the last nucleotide (851st coding exon) of the fourth exon is indicated by the orange square; (**D**) RT-PCR analysis of a testis in *Spata16*^pm/pm^ mice. The 498-bp band was amplified from wild-type and *Spata16*^pm/pm^ mice. A point mutation causing an inappropriate splicing was not found in *Spata16*^pm/pm^ mouse testes; (**E**) Representative testicular histology sections stained with hematoxylin and eosin. Spermatogenesis in *Spata16*^pm/pm^ mice looked normal compared with that in *Spata16*^pm/wt^ mice. Scale bars: 100 μm; (**F**) Cauda epididymal spermatozoa from *Spata16*^pm/wt^ and *Spata16*^pm/pm^ mice. There were no sperm morphology differences between *Spata16*^pm/wt^ and *Spata16*^pm/pm^ mice. *Spata16*^pm/pm^ males were fertile. Scale bars: 50 μm.

**Figure 3:**
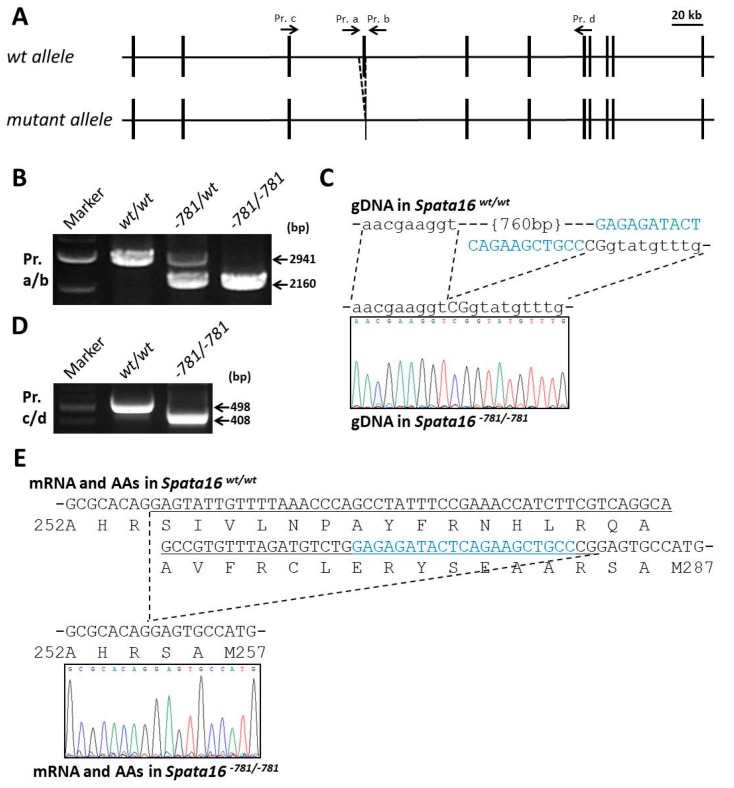
781-bp deletion of *Spata16* by CRISPR/Cas9. (**A**) Targeting scheme of the 781-bp deletion in the mouse *Spata16* locus; (**B**) The genotyping of *Spata16*^−781/−781^ mice by PCR analysis. Both the wild-type allele band at 2941 bp and the deleted allele band at 2160 bp were amplified by PCR; (**C**) Direct sequencing of the 781-bp deletion around the *Spata16* fourth exon. Capital and small letters indicate nucleotides of the exon and intron, respectively. Blue indicates the sgRNA sequence; (**D**) RT-PCR analysis of a testis in *Spata16*^−781/−781^ mice. The 498-bp and 408-bp bands were amplified from wild-type and *Spata16*^−781/−781^ mice, respectively; (**E**) Direct sequencing of *Spata16* mRNAs in wild-type and *Spata16*^−781/−781^ mice. The 781-bp deletion around the fourth exon in *Spata16*^−781/−781^ mice caused the mis-translation of the remaining 2 bp (5′-CG-3′) of the mutant fourth exon. Blue indicates the sequence of the sgRNA.

To study the human point mutation and function of the mouse *Spata16* gene in vivo, we produced a *Spata16* point mutant (851G→A, R284Q) and deleted 781 bp from mice by CRISPR/Cas9 using embryonic stem (ES) cells (Figure 2A and Figure 3A). The *Spata16* point mutant (851G→A, R284Q) and the 781-bp deletion obtained from chimeric males were confirmed by PCR (Figure 2B and Figure 3B) and direct sequencing (Figure 2C and Figure 3C) analyses. An RT-PCR analysis of the *Spata16* point mutant testis showed a single band at 498 bp identical to that of a wild-type testis (Figure 2D). These data indicated that the point mutation in the fourth exon caused no splicing abnormality. The mouse line with the 781-bp deletion removed 88 bp out of 90 bp of the fourth exon of *Spata16* (Figure 3C). This mutation resulted in the mis-translation of the fourth exon encoding the C-terminus of the TPR domain, which was confirmed by the RT-PCR of a *Spata16*^−781/−781^ testis (Figure 3D,E). These results confirmed that we generated a *Spata16* point mutant (851G→A, R284Q, *Spata16*^pm^) and a 781-bp deletion (fourth exon skipped).

### 2.3. Fertilizing Ability of Spata16 Mutant Male Mice

No overt developmental abnormalities were observed in the *Spata16* mutant mouse lines produced. To examine male fertility, adult *Spata16* mutant males were mated with wild-type or mutant females for several months. Whereas the *Spata16*^pm/wt^, *Spata16*^pm/pm^, and *Spata16*^−781/wt^ males were fertile, the *Spata16*^−781/−781^ males were completely sterile despite showing normal mating behavior with successful ejaculation and vaginal plug formation. The mean litter size in *Spata16*^pm/wt^ males was 9.7 ± 0.6 (*n* = 3), 7.7 ± 2.5 (*n* = 3) in *Spata16*^pm/pm^ males, and 9.8 ± 3.3 (*n* = 4) in *Spata16*^−781/wt^ males. The R284Q point-mutant SPATA16 protein caused no deleterious effects on testicular histology (Figure 2E). Spermatozoa produced by *Spata16*^pm/pm^ mice were motile and morphologically normal under phase-contrast microscopy (Figure 2F). *Spata16*^pm/pm^ and *Spata16*^−781/−781^ females were fertile as expected, as *Spata16* is a testis-specific gene (Figure 1A). Although the R284Q point mutation in SPATA16 was not critical for spermatogenesis, SPATA16 was essential for male fertility in mice.

### 2.4. Spermiogenic Arrest in Spata16^−781/−781^ Mice

**Figure 4:**
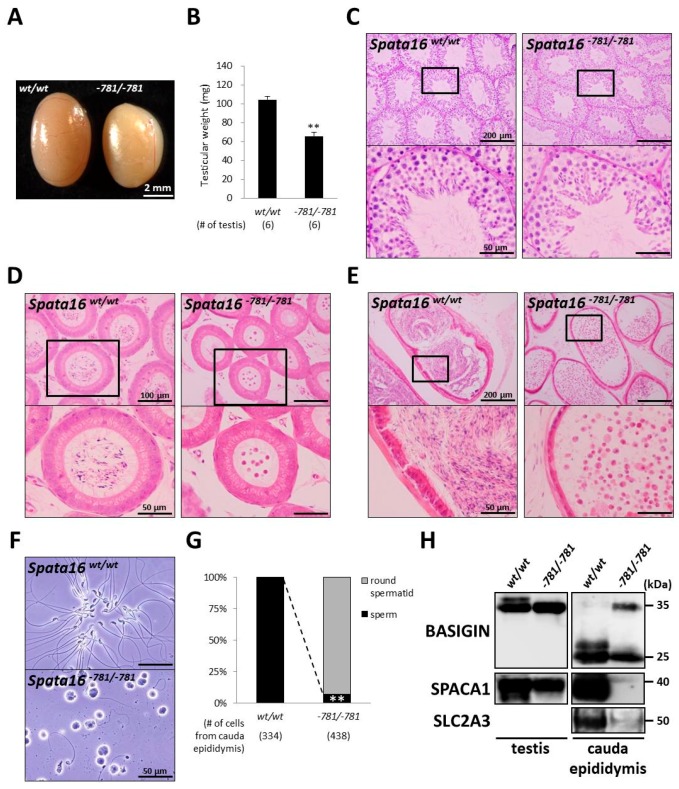
Spermiogenic arrest in *Spata16*^−781/−781^ mice. (**A**) Testes in wild-type and *Spata16*^−781/−781^ mice. Scale bar: 2 mm; (**B**) Testicular weights in wild-type and *Spata16*^−781/−781^ mice. Testicular weights in *Spata16*^−781/−781^ mice were significantly reduced compared with those in wild-type mice. ** *p* < 0.005; (**C**) PAS staining of testicular sections. Lower figures are magnified images of the boxes indicated in the upper figures. Scale bars: upper; 200 μm and lower; 50 μm; (**D**) HE stained sections of caput epididymis. Lower figures are magnified images of the boxes indicated in the upper figures. Scale bars: upper; 100 μm and lower; 50 μm; (**E**) HE stained sections of cauda epididymis. Lower figures are magnified images of the boxes indicated in the upper figures. Scale bars: upper; 200 μm and lower; 50 μm; (**F**) Observation of cells extracted from cauda epididymis. Scale bars: 50 μm; (**G**) Quantitative morphometric analysis of round spermatid and sperm from cauda epididymis. Dashed line indicates a significant reduction in sperm-like cells extracted from the cauda epididymis in *Spata16*^−781/−781^ mice. ** *p* < 0.005; (**H**) Immunoblot analysis of cell lysates collected from a testis and cauda epididymis. Sperm acrosomal protein SPACA1 remained in the *Spata16*^−781/−781^ mouse testis. In cauda epididymal lysates from *Spata16*^−781/−781^ mice, the aberrant retention of testicular BASIGIN was detected and sperm tail-specific protein SLC2A3 and SPACA1 were decreased. *Spata16*^−781/−781^ male mice were sterile because of a spermiogenic arrest. 20 μg of cell lysates were loaded in each lane.

To analyze the infertility of *Spata16*^−781/−781^ males, we observed the testis and epididymis in *Spata16*^−781/−781^ mice. Although the epididymis in *Spata16*^−781/−781^ mice was morphologically normal, the testicular size and weight of *Spata16*^−781/−781^ mice were significantly decreased compared with those of wild-type mice (104.6 ± 3.2 and 65.9 ± 4.4 mg in the wild-type and *Spata16*^−781/−781^ testis, respectively; *p* < 0.005) (Figure 4A,B). To examine the cause of the reduced testicular weight in *Spata16*^−781/−781^ mice, morphological analysis was performed for testicular sections stained by PAS (Figure 4C) and immunofluorescences (Figure S1). Abnormal spermatogenesis was apparent in *Spata16*^−781/−781^ mice (the rate of normal spermatogenesis: 103/103 tubules in the wild-type testis and 30/86 tubules in the *Spata16*^−781/−781^ testis). Observation of the green acrosome derived from the *Acr-Egfp* transgene and the acrosomal membrane protein SPACA1 showed spermiogenic arrest in *Spata16*^−781/−781^ mice (Figure S1). Spermiogenic arrest observed in *Spata16*^−781/−781^ mice likely leads to reduced testicular weight in those mice. Next, to examine the morphology of epididymal spermatozoa in *Spata16*^−781/−781^ mice, we observed epididymal sections stained by hematoxylin and eosin (Figure 4D,E). Although spermatozoa were observed in all tubules of the caput and cauda epididymides in wild-type mice, only a few spermatozoa were detected in those of the caput and cauda epididymides in *Spata16*^−781/−781^ mice. The cells extracted from cauda epididymis in *Spata16*^−781/−781^ mice were observed and classified using morphological and immunofluorescence analyses. Almost all extracted cells were arrested at the round spermatid stage in *Spata16*^−781/−781^ mice (Figure 4F and Figure S2). Seven percent of extracted cells (30/438 cells) were spermatozoa but with abnormal heads and tails, while all cells (334/334 cells) were normal spermatozoa in wild-type mice (Figure 4G). To confirm that the cells were extracted from the cauda epididymis, we performed an immunoblot analysis of three proteins as markers. BASIGIN has been known to change its molecular weight from the testicular size (35 kDa) to the epididymal size (25 kDa) [36]. SPACA1 is as a marker for acrosome biogenesis [31], and SLC2A3 is as a marker for the sperm tail in the epididymis. The cells extracted from the cauda epididymis in *Spata16*^−781/−781^ mice showed aberrant retention of testicular BASIGIN (35 kDa) and the reduction of SPACA1 and SLC2A3 (Figure 4H). Although other KO mice with a globozoospermia-like phenotype showed a significant reduction of testicular SPACA1 [31], SPACA1 remained in the *Spata16*^−781/−781^ mouse testis (Figure 4H). These results indicated that the testicular remnants, including round spermatids, abnormally migrated from the testis to the cauda epididymis in *Spata16*^−781/−781^ mice. The *Spata16* gene in mice, especially the fourth exon encoding the TPR domain, is essential for sperm formation, as revealed by *Spata16* mutant mice.

## 3. Discussion

Globozoospermia is a cause of male infertility characterized by round-headed spermatozoa with an absent acrosome, aberrant nuclear membrane morphology, and sperm midpiece defects [5]. Recently, causative mutations and deletions in three genes, *DPY19L2*, *PICK1*, and *SPATA16*, have been identified from globozoospermic patients [2,3,11,17]. KO mouse experiments also identified seventeen genes responsible for globozoospermia-like phenotypes [4]. Although *Dpy19l2* and *Pick1* have been reported in KO mice, the physiological role of *Spata16* has not been determined.

In this study, we generated CRISPR/Cas9-mediated *Spata16* mutant mouse lines, an 851G→A/R284Q point mutant and a 781-bp deletion. Although globozoospermic patients have a homozygous mutation (848G→A, R283Q) in the fourth exon of *SPATA16* [3], *Spata16*^pm/pm^ (851G→A, R284Q) male mice were fertile. Dam et al.’s paper reported that the 848G→A mutation disrupts the 5′ splicing site of the fourth intron analyzed by in vitro cell transfection experiments and therefore leads to inappropriate splicing of the fourth exon and disruption of the TPR domain of SPATA16. However, in *Spata16*^pm/pm^ (851G→A, R284Q) mice, this mutation did not affect the splicing of the fourth intron (Figure 2D).

In contrast, the *Spata16*^−781/−781^ males were infertile due to spermiogenic arrest, despite 781-bp deletion causing an in-frame mutation of *Spata16* by mis-translation of the fourth exon (Figure 3E and Figure 4). These results suggested that *Spata16*’s fourth exon encoding the C-terminus of the TPR domain is essential for sperm formation and male fertility in mice. In-frame mutations resulting from fourth exon skipping (30 AAs) may also occur in globozoospermic patients with a homozygous point mutation (848G→A, R283Q). Further experiments may be required to examine *SPATA16* mRNA expression in globozoospermic patients with a homozygous *SPATA16* mutation.

We found differences in the phenotype of *SPATA16* mutations between humans and mice. Although the mutation of human *SPATA16* caused globozoospermia with round-headed spermatozoa, *Spata16*^−781/−781^ mice were infertile with spermiogenic arrest, with impaired differentiation of round spermatids into the mature spermatozoa (Figure 4C). In round spermatids, SPATA16 and PICK1 are both localized to the Golgi apparatus [8,19]. *Pick1* KO mice have impaired acrosome formation caused by the abnormal trafficking of acrosomal vesicles from the Golgi to the acrosome [10]. However, *Spata16*^−781/−781^ mice had normal acrosome biogenesis as observed by the green acrosome encoding *Acr-Egfp* transgene and SPACA1 protein remaining in the *Spata16*^−781/−781^ testis (Figure 4H and Figure S1). Testicular SPACA1 is essential for acrosomal formation and is a downstream factor of the other globozoospermia-related proteins [31]. Because the amount of testicular SPACA1 only slightly reduced in *Spata16*^−781/−781^ mice, we can assume that mouse SPATA16 is not related to globozoospermia. Accordingly, mouse SPATA16 is required to progress through the sperm formation, differing from globozoospermic patients with a homozygous SPATA16 mutation. In humans and mice, frameshift mutations and the knockout of *Spata16* will unveil the physiological functions of SPATA16. Moreover, proteins that bind with the SPATA16 TPR domain may imply a key role in spermatogenesis. Thus, to elucidate why patients with SPATA16 mutations show infertility causing globozoospermia, further study of other binding proteins is necessary.

## 4. Materials and Methods

### 4.1. Animals

All animal experiments were approved by the Animal Care and Use Committee of the Research Institute for Microbial Diseases, Osaka University (approval code: H25-02-0; approval date: 21 June 2013). Mice were maintained under a 12-h light/dark cycle. Wild-type mice were purchased from CLEA Japan (Tokyo, Japan) and Japan SLC (Shizuoka, Japan).

### 4.2. RT-PCR

Mouse cDNA was prepared from multiple adult tissues and from one- to five-week-old testes of ICR mice. To confirm the mutation in *Spata16*^−781/−781^ mice, RT-PCR was performed using RNA from a testis. The amplification conditions were 1 min at 94 °C, followed by 30 cycles of 94 °C for 30 s, 65 °C for 30 s, and 72 °C for 30 s, with a final seven-minute extension at 72 °C. The primers used are listed in Table S1.

### 4.3. Protein Sequence Alignment among Mammals

Protein sequence alignment was performed as described previously [37].

### 4.4. Construction of the HDR Donor Plasmid for Point Mutation in Spata16

The *Spata16* gene in mice consists of eleven exons and maps to chromosome 3. The reference plasmid was designed to introduce point mutation in the fourth exon of *Spata16* and was constructed in the pBluescript II SK (+) vector. Homology arms, each 0.5 kb in length, were amplified by PCR using genomic DNA derived from C57BL/6 mice as a template. The primers used are listed in Table S1. The R284Q amino acid substitution of mouse SPATA16 corresponds with the same position (848G→A, R283Q) of globozoospermic patients [3]. This targeted point mutation prevented the genomic re-cleavage after homology-directed repair (HDR).

### 4.5. Generation of Spata16 Mutant Mice with CRISPR/Cas9

*Spata16* mutant mice were produced by the transfection of pX330 plasmids (https://www.addgene.org/42230/) with the HDR donor plasmid into ES cells, EGR-G01 [129S2 × C57BL/6N-Tg(*CAG/Acr-Egfp*)], as described previously [38,39]. A search for single guide RNAs (sgRNAs) and off-target sequences was performed using CRISPRdirect software (https://crispr.dbcls.jp/) [40]. The sgRNA sequence used for transfection was: 5′-GAGAGATACTCAGAAGCTGCC-3′. Screening of ES cell clones was performed by direct sequencing following PCR. The primers used are listed in Table S1. Twenty four clones of transfected ES cells were selected and screened by *Nci*I digestion following PCR. Eight clones contained a disruption at the *Nci*I restriction site and were also confirmed by direct sequencing. Four out of eight clones introduced the targeted point mutation with other indels (insertion/deletion). Three correctly targeted clones were used to make chimeric mice by a microinjection. The mutant ES cell clones were injected into eight-cell stage ICR embryos, and chimeric blastocysts were transferred into the uterine horns of pseudopregnant ICR females the next day. To confirm germline transmission, chimeric males were mated with B6D2F1 females. *Spata16* point mutant mice were genotyped by *Nci*I restriction enzyme (New England Biolabs, Ipswich, MA, USA) digestion following PCR. The *Nci*I recognition site (5′-CCCGG-3′) was disrupted by the point mutation (G to A) at the last nucleotide of the fourth exon. *Spata16* 781-bp deleted mice were genotyped by a 2.9 kb band as the wild-type allele and a 2.2 kb band as the mutant allele.

The *Spata16* mutant mouse lines, STOCK-*Spata16* <em1(R284Q)Osb)> and STOCK-*Spata16* <em2Osb>, were deposited to the RIKEN BioResource Center (http://mus.brc.riken.jp/en/) and the Center for Animal Resources and Development (CARD), Kumamoto University (http://card.medic.kumamoto-u.ac.jp/card/english/).

### 4.6. Male Fertility Test

Sexually mature mutant male mice were caged with two-month-old B6D2F1 or mutant females for several months, and the number of pups in each cage was counted within a week of birth. Average litter sizes are presented as the number of total pups born divided by the number of litters for each genotype.

### 4.7. Testis Weights, Testis Histology, and Sperm Morphology

After breeding studies, males were sacrificed by cervical dislocation following anesthesia. Testes were weighed individually. Testes were fixed in 4% paraformaldehyde in PBS and were processed for paraffin embedding or frozen section. Five micron paraffin sections were stained with periodic acid-Schiff (PAS) and then counterstained with Mayer hematoxylin solution (Wako, Osaka, Japan). Ten micron frozen sections were stained with antibodies for immunostaining. Round spermatids and cauda epididymal spermatozoa were dispersed in PBS, observed under phase-contrast to assess morphology.

### 4.8. Antibodies

The monoclonal antibodies used here were described previously: KS64-10 for SLC2A3 [41]. Polyclonal antibodies were purchased from Santa Cruz Biotechnology (sc-9757 for BASIGIN) and Acris Antibodies (BP5112 for SPACA1). Rabbit antisera against SPACA1 were described previously [31].

### 4.9. Immunostaining

Immunostaining was performed as described previously [42,43]. Briefly, all samples were mounted on glass slides and dried. After washing with PBS, slides were blocked with 10% New Born Calf Serum (NBCS)/PBS for 1 h and incubated with antibodies in 10% NBCS/PBS at 4 °C overnight. After washing with 10% NBCS/PBS containing 0.05% Tween-20, the slides were incubated with secondary antibodies in 10% NBCS/PBS for 1 h. After washing with PBS containing 0.05% Tween-20, the slides were observed under a fluorescence microscope (IX70, Olympus, Tokyo, Japan).

### 4.10. Immunoblot

Immunoblot analysis was performed as described previously [44]. Briefly, testicular germ cells were collected from the seminiferous tubules of testes. Sperm samples were collected from the cauda epididymis and vas deferens. These samples were homogenized in lysis buffer containing 1% Triton X-100 and 1% protease inhibitors (Nacalai Tesque, Kyoto, Japan), and then centrifuged with the supernatants, before being collected. Protein lysates were resolved by SDS/PAGE under reducing condition and transferred to PVDF membranes (Merck Millipore, Burlington, MA, USA). After blocking, blots were incubated with primary antibodies overnight at 4 °C, and then incubated with secondary antibodies conjugated with horseradish-peroxidase. The detection was performed using an ECL plus western blotting detection kit (GE Healthcare, Little Chalfont, UK).

### 4.11. Statistical Analysis

Statistical analyses were performed using a Student’s *t*-test inserted in Excel 2016 (Microsoft, Redmond, WA, USA) after the data were tested for the normality of distribution. Differences were considered significant at *p* < 0.005 (**).

## 5. Conclusions

We have generated a CRISPR/Cas9-mediated mouse model of an *SPATA16* point mutation found in globozoospermic patients. *Spata16* point mutant mice were fertile suggesting that this mutation (851G→A, R284Q) is dispensable for sperm function in mice. This mutation was localized to the fourth exon of the *SPATA16* gene in humans and mice. The replacement of the mouse fourth intron with that of the human *SPATA16* fourth intron by CRISPR/Cas9 may provide the ideal model for the point mutation seen in human globozoospermia (Figure S3). Deletion of the fourth exon of mouse *Spata16* caused male infertility with a spermiogenic arrest. Therefore, *Spata16* itself is indispensable for spermiogenesis and male fertility in mice. Further investigations will be required to clarify the mechanism of SPATA16 during spermiogenesis and the differences in phenotype between humans and mice. These investigations have been aided by CRISPR/Cas9-mediated genome editing, which has emerged as a powerful and efficient method in the biomedical sciences and holds enormous promise for genetic research and therapy in human infertility.

## Supplementary Materials

Supplementary materials can be found at www.mdpi.com/1422-0067/18/10/2208/s1.
