# Supplementary material for: Human Globozoospermia-Related Gene Spata16 Is Required for Sperm Formation Revealed by CRISPR/Cas9-Mediated Mouse Models
Source: Int J Mol Sci. 2017 Oct 21;18(10):2208. doi: 10.3390/ijms18102208 (PMC5666888; doi:10.3390/ijms18102208)
Supplement: Supplementary file 1 [file ijms-18-02208-s001.pdf]

# **Figure S1** (Fujihara et al)

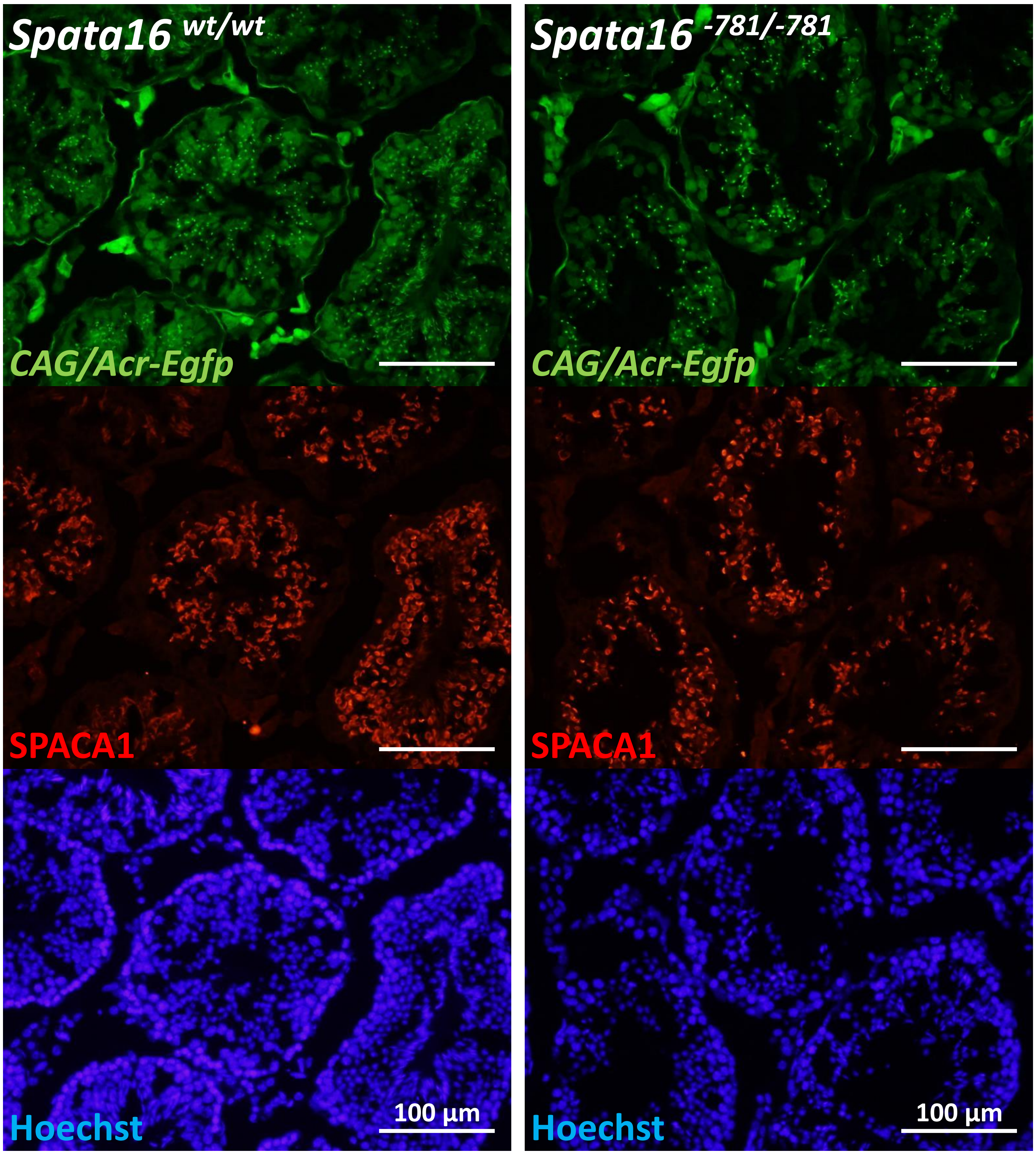


**Figure S1. Histological sections of wild-type and *Spata16*$^{-781/-781}$ testes.**
Acrosomal formation was observed using *CAG/Acr-EGFP* transgenic mouse testis. Red signals indicate the acrosomal membrane protein SPACA1 by immunostaining. Nuclei were stained with Hoechst 33342 (blue). Scale bars: 100 µm.

# **Figure S2** (Fujihara et al)

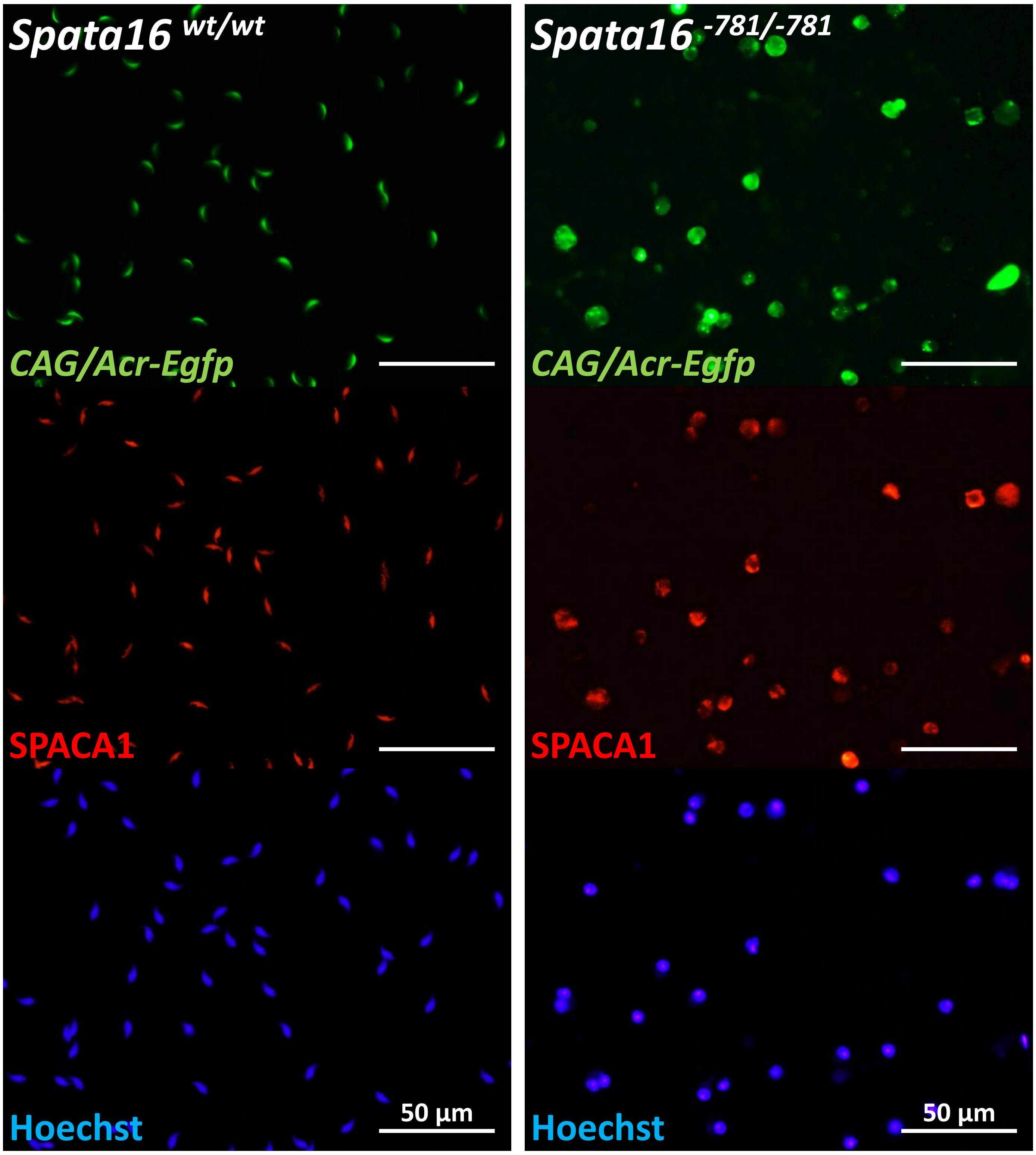


**Figure S2. Observation of extracted cells from cauda epididymis in wild-type and *Spata16*$^{-781/-781}$ mice.**
Acrosomal formation was observed using *CAG/Acr-EGFP* transgenic mouse testis. Red signals indicate the acrosomal membrane protein SPACA1 by immunostaining. Double-positive cells were haploid germ cells with acrosome, round spermatid and spermatozoa. The testicular remnants included round spermatids migrated from testis to the cauda epididymis in *Spata16*$^{-781/-781}$ mice. Nuclei were stained with Hoechst 33342 (blue). Scale bars: 50µm.

# **Figure S3** (Fujihara et al)

```
Human SPATA16 intron4 (1-200)   1 GTATGTTTGTTTTAACTTTTGTGAGGATTTATATCAAGTCAAATTAATTTTATTCTCAAA 60
Mouse Spata16 intron4 (1-200)   1 GTATGTTTGTTACAACTTTC-TGAAGATTTATTTAAAGTTAAATTAATTTTACCCTCAGA 59

Human SPATA16 intron4 (1-200)  61 TTCTGGTAATAGCATAACAGAACTCTCCATTTCTCTCATGGAATATGATTTTATCCAAAT 120
Mouse Spata16 intron4 (1-200)  60 TTCTGGTAATAGCATAAGAGGACATTCCATTTCTCTCATGGAATATGATTTTATCCGAAT 119

Human SPATA16 intron4 (1-200) 121 TATTGTCT-ATCAACGTCAAACCAGCAATTATTGACTATCTTTCTCTTTAATTAAAGCTA 179
Mouse Spata16 intron4 (1-200) 120 TATTGTCTTATAAATGTCAAGCCAACAATTATTGAGTATCTTTTTCTTTAATTAAAGCTA 179

Human SPATA16 intron4 (1-200) 180 TTCCATGTTCATAGCTCTTAA                                        200
Mouse Spata16 intron4 (1-200) 180 TTTCATGTTCATAGCTCTTAG                                        200
```

**Figure S3. Sequence similarity of the fourth intron of SPATA16.**
Sequence alignment was used for the 1st to 200th nucleotides of fourth intron of *SPATA16*. Black indicates a match in humans and mice. 88% of the nucleotide sequence is identical between humans and mice.

# **Table S1** (Fujihara et al)

**Table S1. List of primer sequences**

| Figure | Sequence (5' to 3') | Name |
|---|---|---|
| 1A, 1B, 2D, 3D, 3E | GGAGCAGCTCTGGGGAAACCC | *Spata16*, Pr.c/d |
| | CCAGTCGGCTGTCTGCGGC | |
| 1A, 1B | AAGTGTGACGTTGACATCCG | *Actb* |
| | GATCCACATCTGCTGGAAGG | |
| 1B | GGGAGTCAGTTAGGAGGTCTGATTACAGTAG | *Dpy19l2* |
| | CTGAAATTTGCTTGGCTCAATGTATCCCAC | |
| | GGCTCGAGCCCGCTTCTC | *Pick1* |
| | GCCTTGGTTAGGGCCATAGGC | |
| | GTTATACTGACAAATGACTCAGCAGTCTTGG | *Spaca1* |
| | CATAGAGCTCAGCTCGGACTGTATCTC | |
| 2A | AGGTGAGCGCATGTGCAAGG | 5'-arm |
| | GATATCTCTCCAGACATCTAAACACGG | |
| | GATACTCAGAAGCTGCCCAGTATGTTTGTTACAACTTTCTG | 3'-arm |
| | TCTGGGGCTGAGAACATTAGC | |
| 2B, 3B, 3C | GCCCACTCTGGTTAACTGGGACCC | Pr.a/b |
| | GGGTGCCAAGGACCCAAACTCAG | |
| 2C | CTACGTCAGTGCTGCCAAGTAGC | Point mutation |
| | CCATCATCACTAATGACCTCATGGCTGG | |
